# Late-Onset Hydroxyurea-Induced Melanonychia and Tongue Hyperpigmentation in a Patient With Polycythemia Vera: A Case Report

**DOI:** 10.7759/cureus.53642

**Published:** 2024-02-05

**Authors:** Nena Letete, Deborah Vaz

**Affiliations:** 1 Haematology, Steve Biko Academic Hospital, Pretoria, ZAF; 2 Haematology, National Health Laboratory Service, Pretoria, ZAF; 3 Haematology, University of Pretoria, Pretoria, ZAF

**Keywords:** polycythemia vera, hematology, dermatology, hyperpigmentation, melanonychia, hydroxyurea

## Abstract

In a rare case, a 70-year-old female with polycythemia vera developed late-onset melanonychia, a seldom-documented side effect of hydroxyurea. Typically, melanonychia emerges within months of treatment, but this case is unique as it occurred four years into therapy. Notably, the patient, with darker skin, also had hyperpigmentation of her hands and tongue.

Her history of hydroxyurea-associated ulcers and symptoms worsening with dose adjustment suggested drug involvement. While mucocutaneous hyperpigmentation from hydroxyurea is known, melanonychia and tongue hyperpigmentation are rarely reported, mostly in early treatment.

This case highlights the importance of recognizing these side effects, especially in diverse populations and darker skin tones. The diverse skin tones seen in Sub-Saharan Africa add complexity to diagnosing such dermatological conditions, highlighting the need for awareness. Melanonychia can mimic severe conditions such as subungual melanoma, emphasizing the significance of accurate recognition and management without invasive tests.

Educating clinicians and patients about these benign drug-related phenomena is essential for precise identification and management. This case contributes to understanding late-onset hydroxyurea-induced melanonychia and tongue hyperpigmentation, enhancing clinical knowledge in diverse populations.

## Introduction

Polycythemia vera (PV), a chronic myeloproliferative neoplasm characterized by an elevated red cell mass, is routinely managed with hydroxyurea (HU), an anti-metabolite that selectively inhibits ribonucleoside diphosphate reductase. While hydroxyurea is widely used in PV treatment, its dermatological side effects are well-documented, including skin ulcers and dermatomyositis-like eruptions. However, melanonychia, characterized by brown-black nail discoloration, is rarely described in patients with darker skin. Melanonychia poses a clinical challenge due to its potential association with serious conditions such as subungual melanoma. This case report highlights a unique instance of hydroxyurea-induced melanonychia and hyperpigmentation occurring in a patient with PV, emphasizing the importance of recognizing this infrequently reported adverse effect in clinical practice.

## Case presentation

A 70-year-old female patient presented with gradual hyperpigmentation of nails, hands, and tongue for approximately six months. Her symptoms had been slowly progressive and she endorsed no associated pain, history of trauma, or constitutional symptoms. She reported that the hyperpigmentation had worsened with the recent up-titration of her HU dose.

She had been following up at our Haematology Clinic with PV (JAK2 V617F+) since 2019 and receiving cytoreductive therapy in the form of HU, aspirin, and intermittent venesections since diagnosis, with no history of associated thrombotic events. Her medical history was significant for previous breast cancer for which she underwent a modified radical mastectomy in 2019. She was subsequently treated with anastrozole (an aromatase inhibitor) and remained in remission.

**Figure 1:**
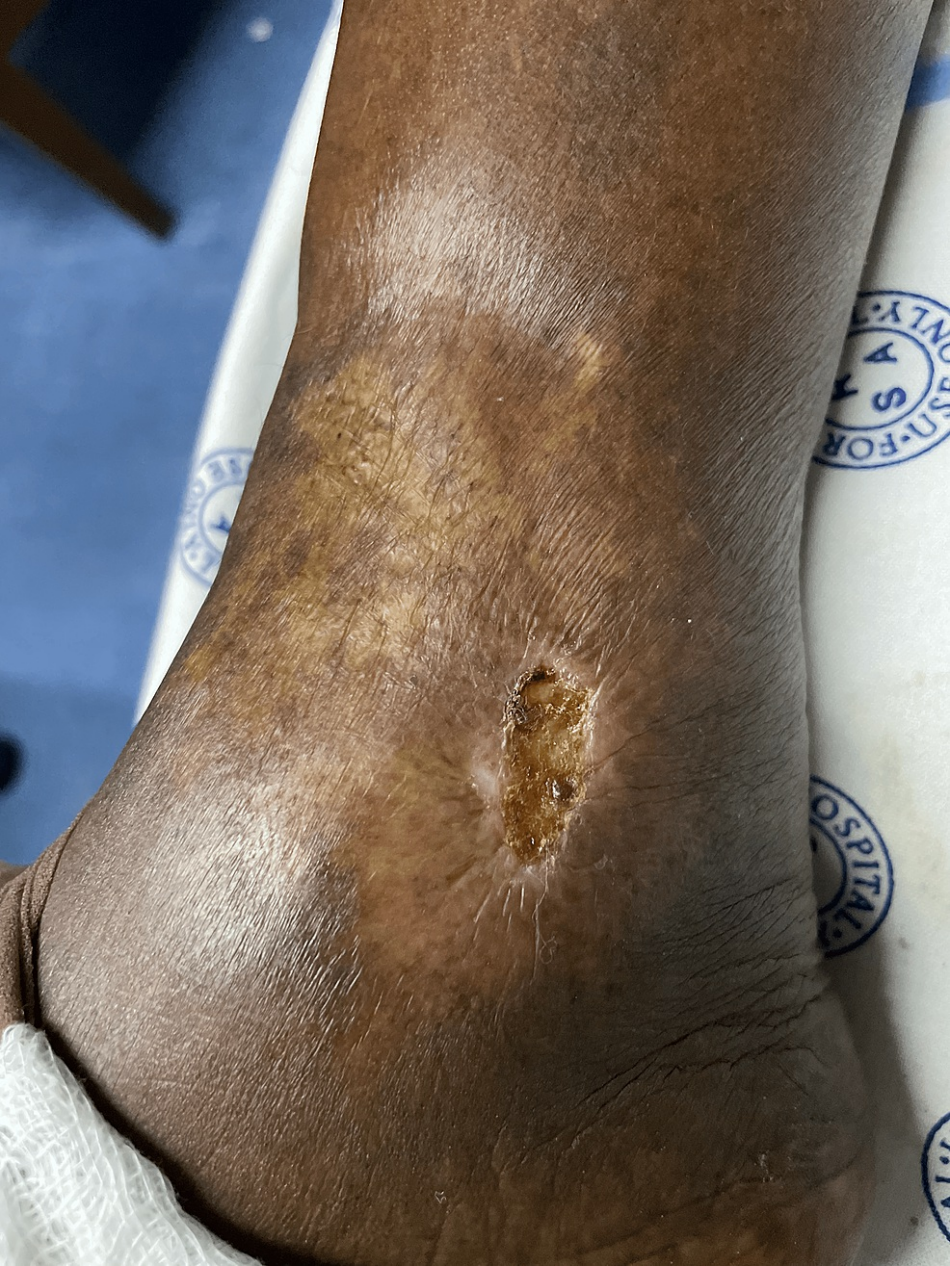
Hydroxyurea-associated right lower limb ulcer measuring 4 x 1 cm shallow and healing.

She was hypertensive on amlodipine and enalapril, complicated with hypertension-mediated organ damage in the form of hypertensive heart disease. Of note, she had a history of HU-associated lower limb ulcers in 2021, a well-defined adverse effect of the drug. Her dosage was reduced from 1,500 mg daily to 500 mg BID and eventually withdrawn after a month, with the resolution of her ulcers (Figure 1). Upon reinitiation of HU at 500 mg BID (15 mg/kg) three months later, she experienced worsening of her ulcers.

**Figure 2:**
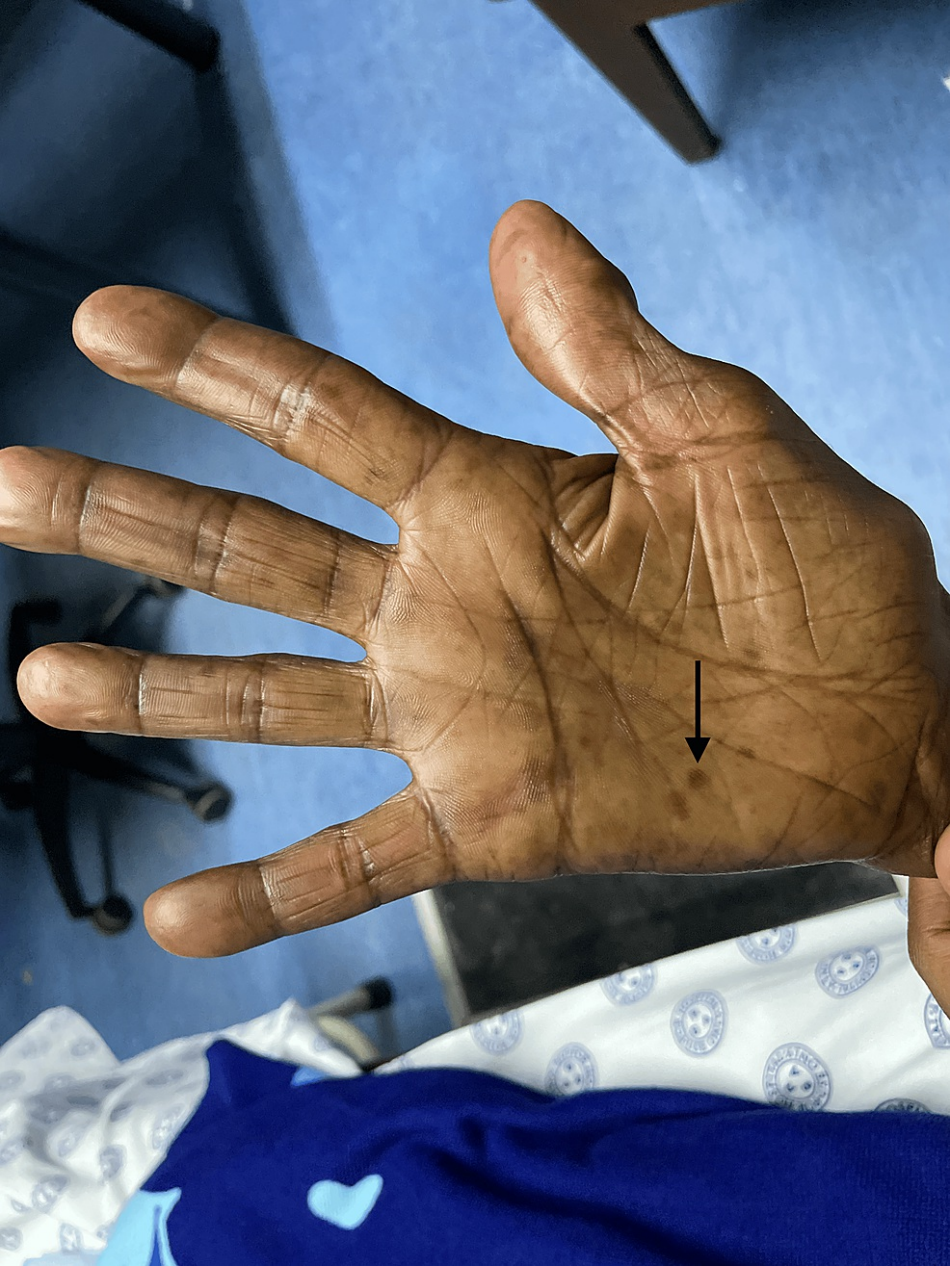
Palmar hyperpigmented macules.

**Figure 3:**
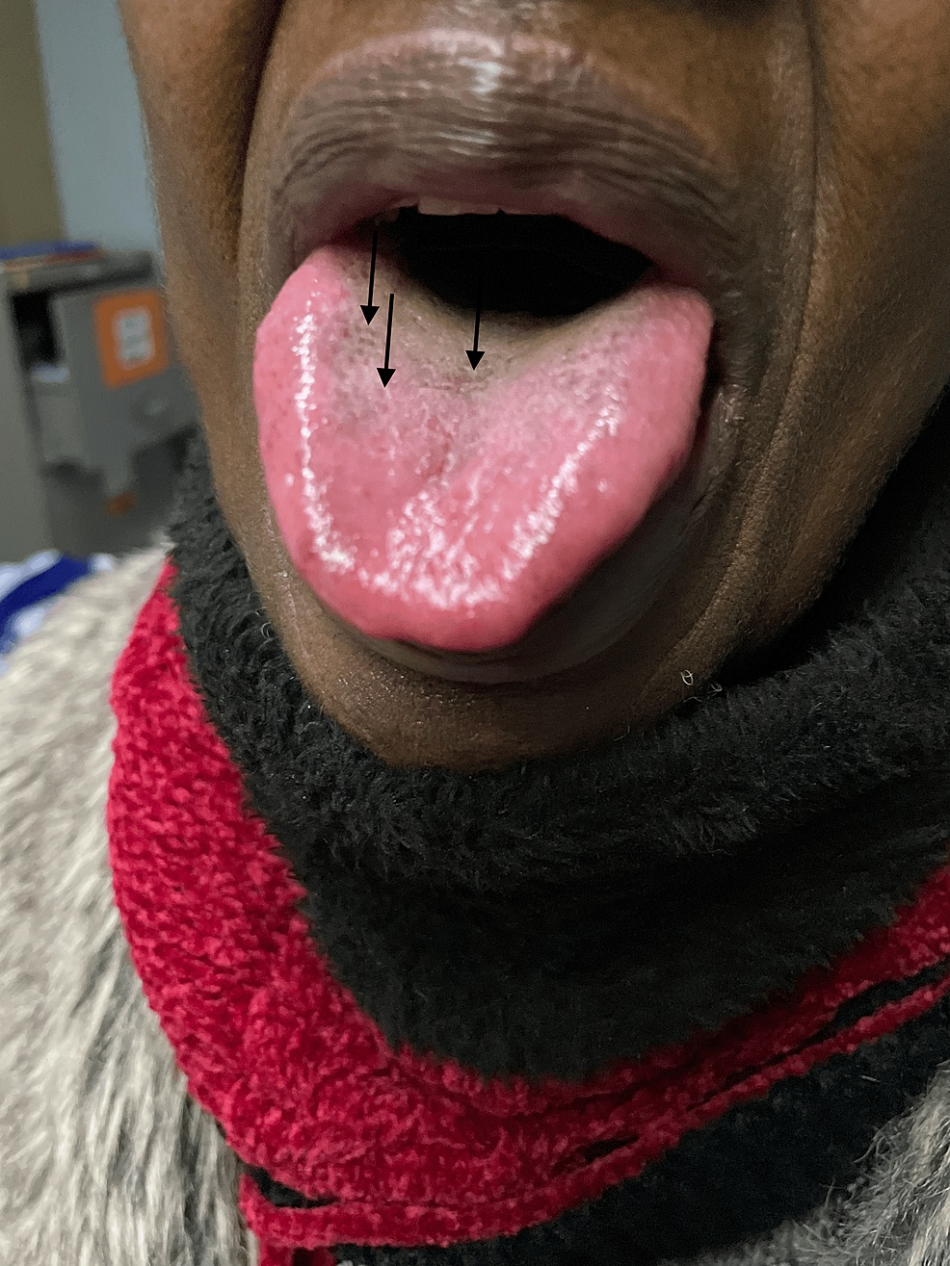
Hyperpigmented lesions on the tongue.

**Figure 4:**
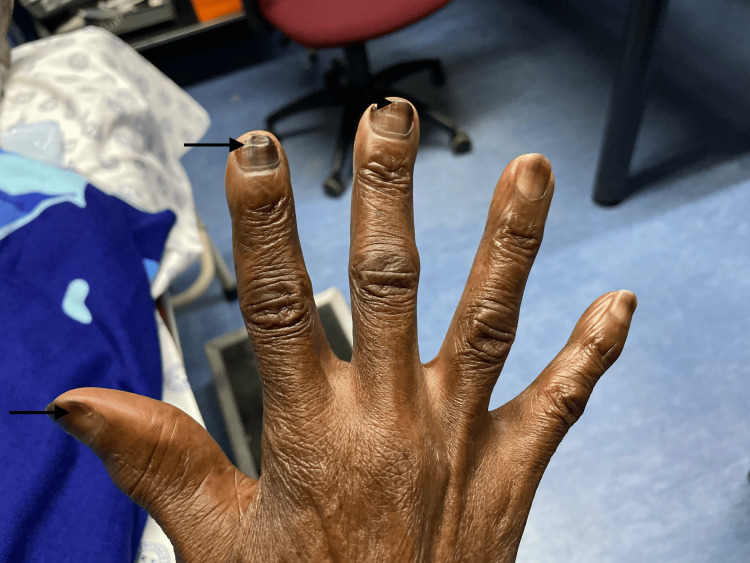
Transverse and longitudinal melanonychia.

**Figure 5:**
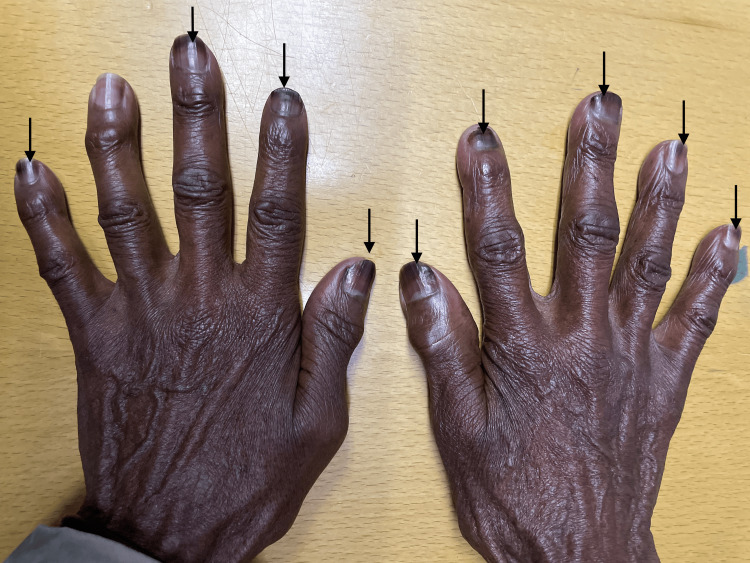
Diffuse, longitudinal, and transverse bands of melanonychia.

On examination, she had Fitzpatrick scale type 6 skin pigment, melanonychia of all 20 nails in her hands and feet (varying from longitudinal and transverse bands to diffuse nailbed involvement), and brown round lesions on the palms of the hands (painless and flat). Similar lesions were also present on the tongue, with multiple, small, round, painless discrete areas of well-circumscribed brown macules (Figures 2-5). The oral mucosa was normal, and no other cutaneous lesions were noted. There was evidence of prior HU-related lower limb ulcers in various stages of healing.

This pattern of hyperpigmentation was initially assessed as fungal melanonychia rather than an idiosyncratic drug reaction and the patient received oral antifungal treatment for seven days, with no improvement. Upon return for review, a decision was made to reduce the dosage of HU to 500 mg once daily. Her lesions improved with reduction of HU dose after two months, and because of a temporal association, melanonychia and hyperpigmentation were attributed to HU.

The absence of suggestive clinical features and having excluded more common causes of hyperpigmentation (Table 1) such as vitamin B12 deficiency, thyroid disorders, hemosiderosis, Addison’s disease, Cushing’s disease, or human immunodeficiency virus also increased the likelihood of a drug-related process.

**Table 1:** Laboratory investigations. TSH: thyroid-stimulating hormone; HIV: human immunodeficiency virus; ELISA: enzyme-linked immunosorbent assay

Analyte	Result	Reference values
Vitamin B12	1117	138–652 pmol/L
TSH	1.24	0.35–4.94 mIU/L
Transferrin saturation	4%	15–50%
9 AM serum cortisol	258	101–536 nmol/L
HIV-1/2 Ab/Ag ELISA	Negative	
Ferritin	25	5–204 µg/L

Treatment, outcome, and follow-up

The dose of HU was reduced due to the cosmetic concerns of the patient. After two months of reduced HU therapy, there was an improvement in nail discoloration with stable hematological parameters. Drug rechallenge led to a recurrence of skin toxicity. Hence, HU was established as the etiologic agent for hyperpigmentation based on the temporal association of the toxicity to the drug, the lack of any other medication used during that time, and the recurrence of the lesions after re-challenge.

In light of the high probability of HU-related adverse drug reaction, a systematic evaluation was undertaken employing the Naranjo scale (Table 2). This scale serves as a valuable tool in assessing potential adverse drug reactions and establishing a causal association. A score of 9 (definite) was calculated, indicating that the reaction exhibited a logical temporal sequence following drug exposure, manifested a recognized response to the suspected drug, demonstrated confirmation through improvement upon drug withdrawal, and could not be reasonably explained by the known characteristics of the patient’s clinical state. Consequently, a causal link was definitively established.

**Table 2:** Naranjo scale (adverse drug reaction probability scale). Definite: ≥9; probable: 5-8; possible: 1-4; Doubtful: ≤0. Available via license: Creative Commons Attribution-NonCommercial 3.0 Unported.

Question	Yes	No	Do not know or not done	Score in our case
Are there previous conclusive reports on this reaction?	+1	0	0	+1
Did the adverse event appear after the suspected drug was given?	+2	-1	0	+2
Did the adverse reaction improve when the drug was discontinued, or a specific antagonist was given?	+1	0	0	+1
Did the adverse reaction appear when the drug was readministered?	+2	-1	0	+2
Are there alternative causes that could have caused the reaction?	-1	+2	0	+2
Did the reaction reappear when a placebo was given?	-1	+1	0	0
Was the drug detected in any body fluid in toxic concentrations?	+1	0	0	0
Was the reaction more severe when the dose was increased, or less severe when the dose was decreased?	+1	0	0	+1
Did the patient have a similar reaction to the same or similar drugs in any previous exposure?	+1	0	0	0
Was the adverse event confirmed by objective evidence?	+1	0	0	0
Total score				9

## Discussion

PV belongs to the BCR-ABL1-negative myeloproliferative neoplasms and is characterized by activating mutations in *JAK2 *and clinically presents with erythrocytosis, variable degrees of systemic and vasomotor symptoms, and an increased risk of both thromboembolic events and progression to myelofibrosis and acute myeloid leukemia. Skin manifestations associated with PV include pruritus, erythromelalgia, and facial plethora [1].

HU was first synthesized in 1869 by Dresler and Stein [2]. It is classified as an anti-metabolite, which selectively inhibits ribonucleoside diphosphate reductase, and in this way prevents the conversion of ribonucleotides to deoxyribonucleotides, thereby halting the cell cycle at the G1/S phase. This process has radiation-sensitizing activity maintaining cells in the G1 phase and interfering with DNA repair [2].

Indications for HU therapy include acute myeloid leukemia for cytoreduction, chronic myeloid leukemia, essential thrombocytosis, PV, sickle cell disease, refractory hypereosinophilic syndrome, and, rarely, head and neck cancers.

In sickle cell disease, the mechanism of action that drives the morbidity and mortality benefit differs slightly. HU increases fetal hemoglobin (HbF) levels by stimulating HbF production, but the exact mechanism of HbF production is unknown. It also has antioxidant properties, alters the red blood cell (RBC) membrane, increases RBC deformability by increasing intracellular water content, and decreases RBC adherence to the endothelium [3].

The more common adverse reactions associated with HU use include macrocytosis, neutropenia with associated bacterial infections, and reduced absolute reticulocyte count with associated anemia.

Skin manifestations of HU treatment that have been described with varying frequency include eczema and xeroderma, which are seen in more than 10% of patients, skin ulcers, oral aphthosis, and dermatomyositis-like eruptions [3]. Mucocutaneous hyperpigmentation is a widely recognized side effect of HU; however, melanonychia is rarely reported with an incidence of <5% and is commonly seen one to two months after initiation. It has been postulated that melanonychia is a consequence of benign melanocyte activation due to the direct toxic activity of the drug [4]. Other important drugs that are reported to cause melanonychia include cyclophosphamide, doxorubicin, lamivudine, zidovudine, and chloroquine [5].

**Figure 6:**
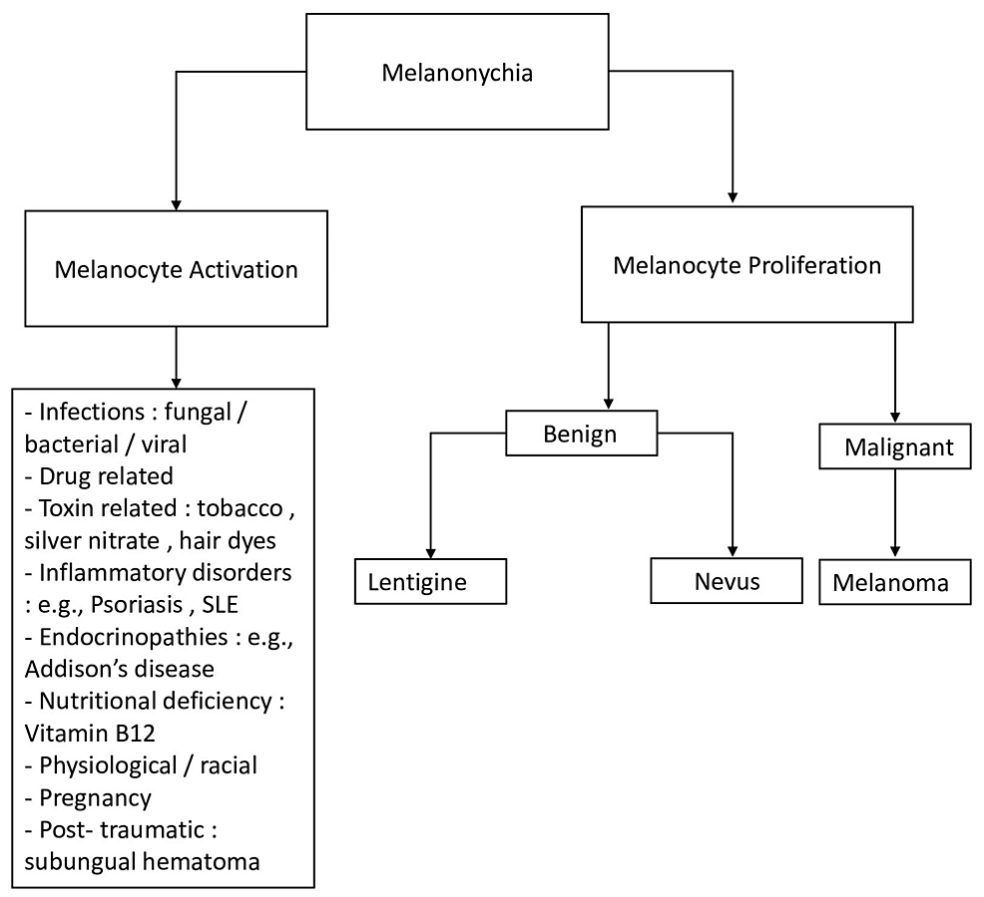
Approach to melanonychia. SLE: systemic lupus erythematosus Image credits: Letete N and Vaz D.

Melanonychia is characterized by brown-black discoloration of the nail plate, typically by melanin, but hematomas may mimic this clinical picture [5]. Melanocytes are present in both the nail matrix and bed but are often dormant. Melanocyte activation of a normal number of activated melanocytes occurs and they migrate to the nail plate and cause visible pigmentation [5]. This accounts for the majority of causes of melanonychia and includes pregnancy, infections, drugs, endocrinopathies, and inflammatory disorders (Figure 6).

The second mechanism entails nail matrix melanocyte proliferation, that is, an increased number of melanocytes in the nail matrix. This proliferation may be benign (lentigine or nevus) or malignant (melanoma).

The most common cause of melanonychia in a Korean case series was reported to be subungual hemorrhage (29.1%), followed by nail matrix nevus (21.8%), trauma (14.5%), lentigine (11.6%), and racial melanonychia (8.0%) [6]. Typical nail involvement is longitudinal and diffuse melanonychia hyperpigmentation. This clinical picture may lead to the misdiagnosis of a malignancy (subungual melanoma/pigmented squamous cell carcinoma). Single nail involvement with co-existent Hutchinson’s sign (periungual extension of pigmentation from longitudinal melanonychia onto proximal and lateral nail folds) would be more suggestive of melanoma [7].

Aste et al. reported nail hyperpigmentation in nine patients (out of a cohort of 210 patients) appearing between 6 and 24 months of HU therapy, with the most common presentation being longitudinal melanonychia [8]. They reported the co-occurrence of both melanonychia and skin hyperpigmentation was an even rarer phenomenon, with only a single patient in their cohort experiencing this constellation of symptoms. Furthermore, the late onset of this phenomenon is exceedingly rare, as only one case series has highlighted this. Delmas-Marsalet et al. documented three cases of late-onset (2.5 to 5 years) in 1978 [9].

HU-induced melanonychia has not only been described in the adult population but also in pediatric patients. Montalembert et al. reported five cases (in a cohort of 35 children) receiving HU for the management of sickle cell disease who developed melanonychia in the five years of observation, highlighting the importance of recognition across patient populations [10].

**Figure 7:**
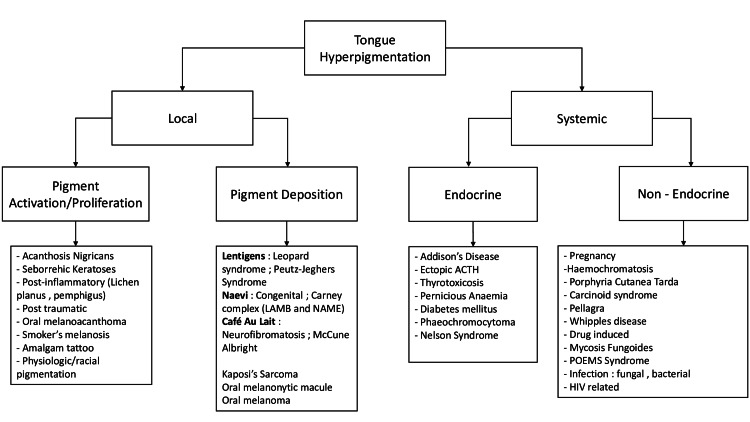
Approach to tongue hyperpigmentation. LAMB: lentigines, atrial myxoma, mucocutaneous myxoma, blue nevus syndrome; NAME: nevi, atrial myxomas, myxoid tumors, ephelides; ACTH: adrenocorticotropic hormone; POEMS: polyneuropathy, organomegaly, endocrinopathy, monoclonal gammopathy, skin changes Image credits: Letete N and Vaz D.

Oral hyperpigmentation is a commonly described adverse effect of hydroxyurea; however, involvement of the tongue is often overlooked. Clinicians must be aware of this infrequently documented phenomenon, as it may not only reflect drug toxicity but may reflect a systemic disease process (Figure 7) [11].

## Conclusions

In the context of HU-associated side effects, melanonychia is a phenomenon that is rarely described in the literature and typically emerges within months of treatment initiation. This report, however, brings to light an extraordinary case of a 70-year-old female with PV and a history of breast cancer who developed melanonychia four years after starting HU therapy.

The delayed onset of melanonychia introduced diagnostic complexities, particularly within the context of Sub-Saharan Africa. Sub-Saharan Africa is characterized by a wide range of skin colors and pigmentation, reflecting the diversity of skin tones. This diversity can sometimes pose challenges in identifying and diagnosing dermatological conditions such as melanonychia, as the presentation may differ based on an individual’s skin color. The challenges in diagnosing dermatological conditions in individuals with darker skin colors stem from limited reference material, under-recognition, and a scarcity of published literature documenting unique presentations of such ailments in the African population. Limited healthcare resources and potential underreporting of infrequent presentations further contributed to the difficulties in diagnosing and managing this unusual presentation.

Notably, her history of HU-related ulcers and the exacerbation of symptoms upon dose adjustment bolstered the argument for drug-induced causality. While mucocutaneous hyperpigmentation due to HU is well recognized, melanonychia and tongue hyperpigmentation remain rarely described phenomena, often manifesting within the initial months of treatment. It is important to note that although it remains rarely described in the literature, the true incidence of these adverse effects is not known and likely underestimated. It is also important to note that anastrozole has been associated with cutaneous vasculitis, erythema multiforme, and acne eruptions, and the skin toxicities described in this case report are not consistent with this drug.

This unusual presentation emphasizes the need to consider the possibility of such late-onset HU-induced melanonychia and hyperpigmentation in patients of African descent. It also highlights the importance of educating both clinicians and patients about these potential adverse effects. By doing so, accurate recognition and appropriate management can occur without unnecessary invasive testing.
